# Effect of Sleep Hygiene on Metabolic Syndrome in an Adult Rural Population in India: Protocol for a Cross-Sectional Study

**DOI:** 10.2196/90117

**Published:** 2026-10-20

**Authors:** Rishabh Kothari, Sonali Choudhari

**Affiliations:** 1 Department of Community Medicine Jawaharlal Nehru Medical College Datta Meghe Institute of Higher Education and Research Wardha, Maharashtra India

**Keywords:** metabolic syndrome, sleep hygiene, rural India, cardiometabolic risk, cross-sectional study, hypertension, hyperglycemia

## Abstract

**Background:**

Metabolic syndrome (MetS) refers to a group of conditions including high blood pressure, excess weight, high blood glucose, and abnormal cholesterol and triglyceride levels that together substantially increase the risk of heart disease and diabetes. Poor sleep hygiene is associated with a higher likelihood of type 2 diabetes, while sleep duration can affect the development of various MetS components. Approximately 25% of adults worldwide currently have MetS, and this prevalence is projected to increase to 50% by 2035. However, there is a distinct lack of evidence about the specific impact of sleep hygiene on cardiometabolic health within the rural Indian population. This study will focus on addressing this literature gap by evaluating these risk factor associations by using validated tools to help develop targeted community interventions.

**Objective:**

This study aims to estimate the prevalence of poor sleep quality and cardiometabolic risk factors in a rural Indian population and to determine the association between sleep hygiene and cardiometabolic risk factors.

**Methods:**

This study will be a community-based, cross-sectional, analytical study, and data collection will be carried out by trained personnel using validated tools during house-to-house visits. In this cross-sectional study of 281 rural Indian adults aged ≥40 years, sleep quality will be assessed via the Pittsburgh Sleep Quality Index (a score >5 indicates poor sleep), alongside direct measurements of blood pressure, fasting plasma glucose, BMI (≥25 kg/m² for obesity), waist circumference, and the Madras Diabetes Research Foundation-Indian Diabetes Risk Score for diabetes risk. Data will be collected through a door-to-door approach, with informed consent entered into Google Forms, and quality checked via training and regular reviews. Using R software, analyses will include prevalence estimates with 95% CIs; chi-square or *t* tests for associations between poor sleep and cardiometabolic risks; and logistic regression (*P*<.05) adjusted for confounders such as diet, stress, and physical activity.

**Results:**

This study began in January 2025 with conceptualization and ethics approval obtained in June 2025. Data collection commenced in August 2025 following Clinical Trial Registry–India registration, with 53 participants enrolled as of May 5, 2026. Standardized tools (GAD-7, PHQ-9, and GPAQ) were incorporated to assess key confounding variables. Data collection is projected to finish by January 2027, with final analysis and study conclusion expected by March 2027.

**Conclusions:**

The relationship between MetS and sleep hygiene warrants greater attention, particularly as poor sleep habits become increasingly prevalent alongside MetS. This study will deepen the understanding of their connection, paving the way for targeted interventions and prevention strategies.

**Trial Registration:**

Clinical Trial Registry–India CTRI/2025/08/092892; https://tinyurl.com/3sak2h9x

**International Registered Report Identifier (IRRID):**

DERR1-10.2196/90117

## Introduction

### Background

Sleep is a fundamental component of a healthy life and is essential for maintaining optimal physical and mental well-being, thereby reducing the risk of fatigue-related accidents and injuries. Sleep duration has been identified as a risk factor for developing metabolic syndrome (MetS) and a potential link to type 2 diabetes [1].

Globally, the prevalence of MetS in the adult population is approximately 25%, with substantial regional variance (33.4% in the United States, 24% in Asia, and 14.4% in China). Projections estimate that MetS prevalence could reach approximately 53% globally by 2035 [2]. MetS has been defined by the American Heart Association and National Heart, Lung, and Blood Institute as the presence of any 3 of 5 risk factors in Asian populations, namely high blood pressure, central obesity, high fasting plasma glucose (FPG), high triglycerides, and low levels of high-density lipoprotein (HDL) [3,4].

Sleep and its role in MetS is a complex relationship. Individuals with shorter sleep durations (<6-7 hours) or longer sleep durations (>8-9 hours) were seen to have a higher prevalence of MetS than those with a normal sleep duration (7-8 hours). Notably, both short and long sleep durations have been associated with an increased risk of different MetS components, suggesting distinct pathophysiological pathways [3]. Overall, sleep duration is associated with an increased risk of developing various MetS components. Another study noted that although it has been concluded that there is a correlation between blood lipid levels and sleep quality, the findings showed inconsistencies, which were attributed to geographical variation, differences in sample sizes, and varying confounding factors [1,5].

Inconsistencies in various studies on sleep and its relation to MetS have been observed. For instance, a study noted that although evidence exists for a possible link between sleep and MetS, findings have been inconsistent, with some studies suggesting that long sleep duration is not associated with MetS development [6]. In contrast, another case-control study, consisting of 2686 participants, concluded that longer sleep durations (>7 hours per day) were associated with risk factors of MetS [1]. Yet another cross-sectional study, conducted on the adolescent population of São Luís with a final sample size of 1268 adolescents within the age range of 18 to 19 years, concluded that there was no association observed between sleep duration and cardiometabolic outcomes in the adolescent population [7]. A meta-analysis found that average sleepers, defined as those who slept 6 to 8 hours per day, had the highest prevalence of MetS (52% of the study population), whereas long sleepers, defined as those who slept >8 hours per day, had a lower prevalence of MetS at 15%. The participants in this analysis ranged in age from 10 to 80 years [2].

The present inconsistencies warrant further research into sleep and its relationship with MetS. This study looks at sleep hygiene, which includes various behavior and lifestyle interventions that can influence a person’s sleeping habits and sleep duration, and its relation to the presence of MetS or its components in an individual. Studies conducted in rural Indian populations have found that, although the prevalence of MetS is lower in rural than in urban populations, it has been increasing in rural areas, raising concerns about undiagnosed health disparities [8]. A study conducted in a rural hospital in Goa, India, on a diabetic population found significant association of poor sleep hygiene and type 2 diabetes mellitus in the study population [9], while another study conducted in Uttarakhand, India, found significant association between sleep hygiene and participants with hypertension [10]. The recent literature has demonstrated that even in the rural population, there has been a rise in MetS, while also showing a clear association between components of MetS and sleep hygiene. There appears to be a critical gap in knowledge in this area. Therefore, this study aims to determine whether poor sleep hygiene, compared with good sleep hygiene, increases the risk of cardiometabolic abnormalities (central obesity, hypertension, and hyperglycemia) among adults aged ≥40 years in a rural Indian population.

### Objectives

This study aims to (1) estimate the prevalence of poor sleep quality among the rural Indian population using the Pittsburgh Sleep Quality Index (PSQI), (2) estimate the prevalence of cardiometabolic risk factors among the rural Indian population, and (3) determine the association between sleep hygiene and cardiometabolic risk factors.

## Methods

### Study Design

This community-based, cross-sectional, analytical study on the effect of sleep hygiene on cardiometabolic health among the adult population commenced in 2025, focusing on individuals in Seloo Taluka, Wardha, Maharashtra. Trained personnel will conduct door-to-door house visits to identify eligible participants and collect data from those who meet the specified eligibility criteria. Blood samples will be collected to measure FPG, anthropometric measurements (height and weight) will be obtained to calculate BMI, waist circumference will be measured as an indicator of obesity [11,12], and blood pressure will be recorded. Questionnaires will be used to assess participants’ sleep hygiene across 7 broad domains, namely subjective sleep quality, sleep latency, sleep duration, habitual sleep efficiency, sleep disturbances, use of sleep medication, and daytime dysfunction. To facilitate participant cooperation, adequate time will be allocated for data collection according to participants’ needs. Participants will also be provided with the investigators’ contact information and may call them with any questions or concerns.

### Study Setting

The study setting will be the field practice area (rural community) of the department or institution in Seloo Taluka. Within the study area, blocks will be identified within the selected villages, and households will be approached sequentially, starting from the market area and proceeding according to house numbers. Each block will be covered completely before proceeding to the next block, and this process will continue until the entire village has been covered. This process is being followed to ensure that all households are covered in an orderly manner. Any households that are missed due to household numbering or other problems will be accounted for after a round of collection has been completed in the block using the abovementioned method.

### Recruitment

To include participants in an age group in which the effects of sleep hygiene on cardiometabolic health are more evident, individuals aged ≥40 years will be enrolled in this study. A robust study conducted by Confortin et al [7] demonstrated no significant relationship between sleep hygiene in adolescents and MetS, while another study conducted in northern India found MetS to be more prevalent in people aged >40 years [13]. Another study conducted in urban and rural south India noted that the age of ≥40 years was identified as a significant predictor of MetS [14], which has provided further rationale to the selection of the abovementioned age group. Participants in this age group will be required to fill an informed consent form, which will be explained to them in their local language, following which the process of data collection will begin. All individuals who have been diagnosed with a sleep disorder (eg, obstructive sleep apnea, narcolepsy) or have existing severe morbid conditions (eg, recent myocardial infarction, advanced diabetes complications, end-stage renal disease, cancer, severe depression) will be considered ineligible for the study and excluded from participation.

### Study Duration

The study will be conducted over 2 years, from January 2025 to January 2027.

### Sample Size

The sample size has been calculated based on a prior study, which noted that the prevalence of MetS was 24% (prevalence [p]=24%) in Asia [2], with a CI of 95%, type 1 error being 5% (α=.05), where *Z*=1.96, and considering a standard margin of error as 5% (*d*=0.05). The required minimum sample size is 281 [2,15], which is calculated as follows:

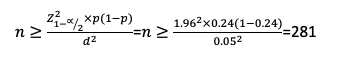



### Sampling Method

This study will use a stratified random sampling method to ensure that different groups within the village are fairly represented. The sample will be selected in 2 stages: first by ward and then by gender, so that both the location and the gender of participants are systematically considered.

First, all wards in the village will be identified and treated as the main strata for sampling. Within each ward, the population will then be divided by gender to maintain a balanced number of male and female participants in the study. An equal number of male and female participants will help ensure that the study is adequately powered to detect any gender-specific differences.

Data collection in each ward will start from the center and proceed outward until the required number of participants for that ward has been reached. The same sampling and data collection procedure will be applied consistently across all wards in the village to keep the process uniform and reliable. The sample size of 281 will be distributed proportionally among the wards according to their respective population sizes. In the event of nonresponse or refusal, the adjacent eligible household will be considered.

### Ethical Considerations

#### Institutional Ethics Committee

The study protocol was approved by the institutional ethics committee of Datta Meghe Institute of Higher Education and Research (DMIHER(DU)/IEC/2025/434) and was registered prospectively in the Clinical Trials Registry–India (CTRI/2025/08/092892).

#### Consent

Written informed consent will be obtained from all participants after explaining the study’s purpose, procedures, benefits and potential risks, voluntary participation, and their right to withdraw at any time.

#### Confidentiality and Privacy

Participants’ personal information will remain confidential, and data will be anonymized for analysis.

#### Participant Compensation

No participant compensation will be provided.

### Data Collection Instruments or Tools

#### PSQI

The PSQI was designed to assess sleep quality in populations with psychiatric disturbances and has been well established as a self-administered questionnaire for evaluating sleep quality. Broadly, the questionnaire covers subjective sleep quality, sleep latency, sleep duration, habitual sleep efficiency, sleep disturbances, use of sleep medication, and daytime dysfunction, while also covering the respondent’s roommate’s responses, although these responses are not scored. There are open-ended and Likert-type questions in the questionnaire, where each question has a score ranging from 0 to 3, with the total possible score being between 0 and 21. A global PSQI score of more than 5 indicates poor sleep quality, regardless of the age group [16,17].

#### Evaluation of MetS Risk Factors Through Measurement of Blood Pressure, Blood Glucose, and Central Obesity

MetS is a group of conditions that increases the risk of heart disease. It is diagnosed based on the presence of factors such as central obesity, high blood pressure, elevated blood glucose and triglyceride levels, and low levels of HDL cholesterol [4]. In this study, cardiometabolic risk will be specifically evaluated based on 3 measurable parameters: high blood pressure, high blood glucose, and central obesity. Triglyceride and cholesterol levels will not be measured because of financial and logistical constraints. The presence of all 3 factors in an individual classifies them as having MetS. Blood pressure will be measured after the participant has rested calmly in a supine position for 10 minutes. Two readings will then be recorded 5 minutes apart using an appropriately sized cuff placed on the right arm. Blood pressure will be measured using a mercury-free blood pressure apparatus (Diamond BPD G-041). Hypertension is defined as systolic blood pressure (SBP) of 130 mm Hg or higher or diastolic blood pressure (DBP) of 80 mm Hg or higher, while individuals with persistent blood pressure of 140/90 mm Hg will be considered for treatment, with a target blood pressure of 130/80 mm Hg or lower [18]. For blood glucose, fasting capillary blood glucose readings will be obtained through a finger prick, measured through a blood glucose meter (NoCoding1 Plus, Model GM01BAA, i-SENS, Inc). An FPG level of ≥126 mg/dL is considered diagnostic of diabetes [19]. Abdominal obesity (central obesity), which is defined as a waist circumference of ≥90 cm for male participants and ≥80 cm for female participants [20], will be assessed using a measuring tape (150 cm; New Trend) to the nearest 0.1 cm, with the waist circumference measured midway between the lower ribs and the iliac crest. Along with waist circumference, BMI will be calculated for obesity classification, with a BMI of ≥25 kg/m^2^ (calculated as weight [kg] divided by height [m²] [21,22]) classified as obese. Height will be measured in meters using a stadiometer (Precision Model, PrimeSurgicals), with participants standing upright and barefoot with their feet together and heels and shoulders touching the vertical rod. Weight will be measured in kilograms using a weighing scale (ActiveFlex LLWS45; Lifelong), with participants standing barefoot and still on the scale.

#### Madras Diabetes Research Foundation

The Madras Diabetes Research Foundation-Indian Diabetes Risk Score is a tool developed for the assessment of type 2 diabetes in the community. It assesses 4 risk factors, namely age, abdominal obesity, physical activity, and family history, yielding a score of up to 100. A score of <30 indicates low risk, 30 to 50 indicates medium risk, and ≥60 indicates high risk of developing type 2 diabetes [23].

### Data Collection Procedure

The study team will visit potential participants at their homes using a door-to-door approach, beginning from the center of each ward in the village and then systematically moving outward to cover the surrounding households. This approach is intended to ensure that residents across the ward are given an equal opportunity to participate, while also making data collection practical and organized (Figure 1).

Before any data are collected, the purpose and procedures of the study will be explained to each eligible participant in clear and simple terms, and a written or verbal informed consent will be obtained in line with ethical guidelines. Once consent is obtained, participants will be interviewed in their preferred language to ensure that they fully understand the questions and can respond accurately without language barriers. Because of resource and time constraints, the study will focus on the following measurements and tools: sleep quality assessment using the PSQI; sleep hygiene assessment using the Sleep Hygiene Index (SHI) scale [24]; and height and weight measurements for calculating BMI, waist circumference, blood pressure, and FPG levels. Together, these parameters will be used to determine whether the participant meets the criteria for MetS, with the presence of any 3 of the 5 criteria indicating MetS. This will help in providing an insight into the cardiometabolic health status of the participant.

All responses obtained for the questionnaire and the measured values will be entered into a structured Google Form at the time of data collection. The benefit of using a digital data entry platform is that it would help minimize errors associated with manual recording, facilitate secure storage of information, and streamline the organization and analysis of data at later stages of the study.

**Figure 1:**
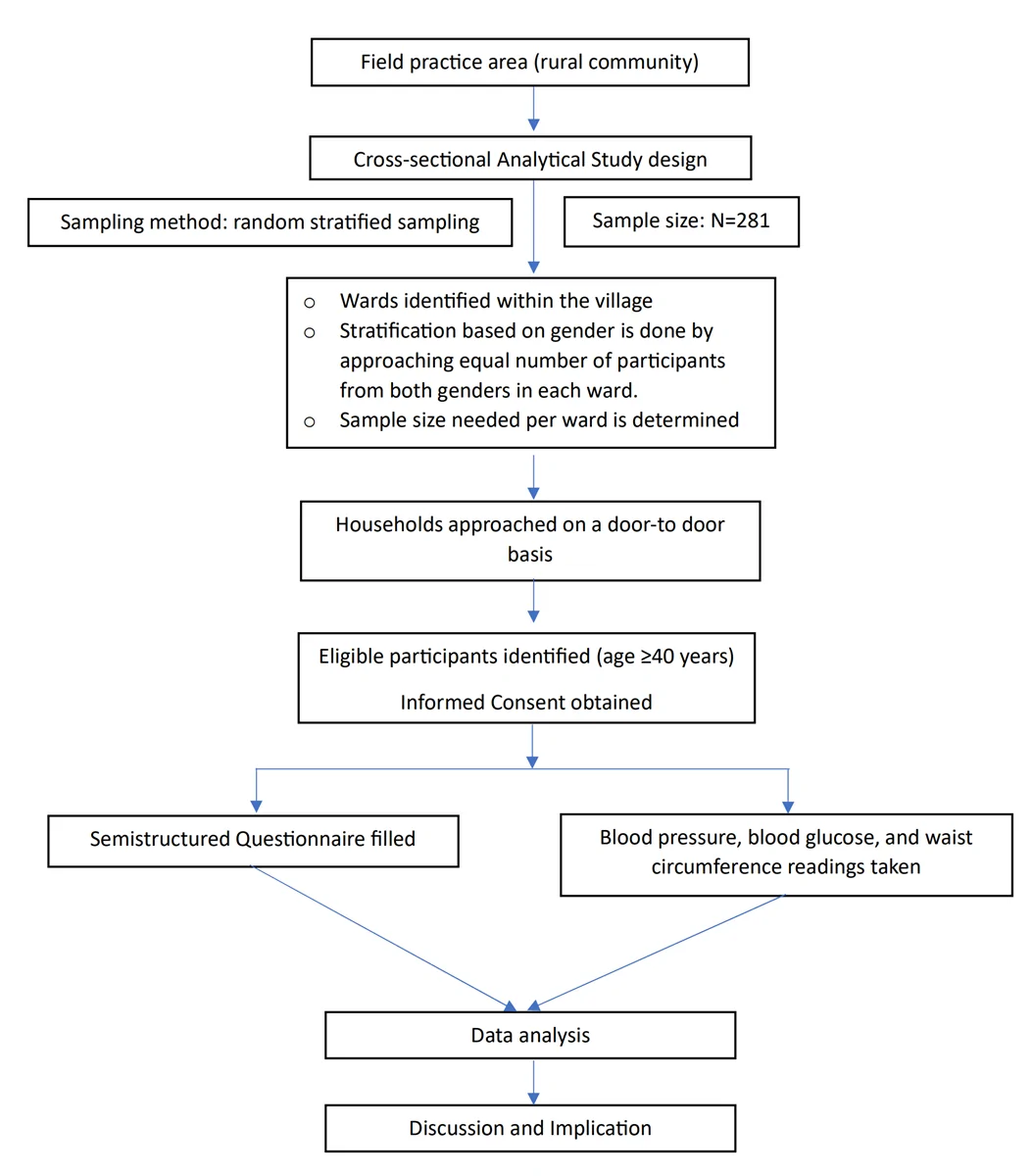
Flowchart depicting data collection and analysis.

### Data Quality Control and Management

In this study, data quality will be maintained through standardized data collection procedures, trained personnel, and the use of pretested tools and validated instruments. Care will be taken that data are collected in a consistent manner across various instances of data collection. Data entry will be regularly monitored for consistency, completeness, and accuracy, with periodic audits and cross-checks to minimize errors and biases. Any discrepancies identified will be promptly addressed through verification with original sources. Additionally, digital tools with built-in validation checks may be used to further enhance data integrity. This systematic approach will be followed stringently to ensure that the findings of the research are credible, valid, and reproducible.

### Operations Terms: Definitions

To ensure standardization and reproducibility across the study protocol, key operational variables and diagnostic thresholds are defined below:

#### Sleep Hygiene

Sleep hygiene refers to behavioral and lifestyle interventions around sleep time and throughout the day that positively influence sleep quality and duration [25]. Sleep quality and duration can be measured by the PSQI [3,16]: (1) 19 items will be used to generate a 7-component score; (2) a higher score indicates poorer sleep quality; and (3) the sum of the 7-component score will produce a global score between 0 and 21 points, where a score >5 has diagnostic sensitivity of 89.6% and specificity of 86.5%.

#### MetS

MetS is defined according to the statement of the American Heart Association and National Heart, Lung, and Blood Institute for Asian populations. A participant will be considered to have MetS when any 3 of the following 5 risk factors are present [3]: (1) waist circumference ≥90 cm for men and ≥80 cm for women; (2) SBP ≥130 mm Hg, DBP ≥80 mm Hg, or current medication for hypertension; (3) FPG ≥5.6 mmol/L (≥126 mg/dL) or current use of antidiabetic therapy; (4) HDL-cholesterol <1.03 mmol/L in men and <1.3 mmol/L in women; and (5) triglyceride ≥1.7 mmol/L.

### Data Analysis Plan

#### Data Management

All data collected during the study will be organized and initially stored using Microsoft Excel, which provides a reliable and accessible platform for entering, sorting, and preliminary cleaning of the dataset before more advanced analyses are performed.

#### Statistical Software

The primary software for conducting the statistical analyses will be R for Windows (version 4.5.2; R Foundation for Statistical Computing) chosen for its robust capabilities in handling epidemiological data, producing high-quality visualizations, and executing complex statistical models with open-source reproducibility.

#### Descriptive Statistics

Descriptive statistics will be applied to summarize key demographic variables and baseline characteristics of the study participants. This will include calculations of means and SDs for continuous measures, as well as frequencies and percentages for categorical data, offering a clear overview of the sample composition and initial patterns in the data.

#### Prevalence Assessment

To determine the prevalence of poor sleep hygiene alongside cardiometabolic risk factors, simple descriptive analyses will be conducted, supplemented by the computation of proportions along with their corresponding 95% CIs. These estimates will provide precise measures of occurrence within the village population, accounting for sampling variability and enabling reliable population-level inferences.

#### Association Testing for Categorical Variables

The chi-square test will be used to examine associations between categorical variables such as poor sleep hygiene and the presence of cardiometabolic risk factors. This nonparametric test is well suited for epidemiological research involving nominal data, as it evaluates whether observed frequencies differ significantly from those expected under independence, helping to identify potential links without assuming normal distributions.

#### Group Comparisons for Continuous Variables

For continuous cardiometabolic parameters, including blood pressure and FPG, the independent *t* test or Mann-Whitney *U* test will be selected based on data normality, which will be assessed through visual methods such as histograms and *Q*-*Q* plots. The *t* test assumes normality and equal variances for comparing means between groups with poor sleep hygiene and those without, while the Mann-Whitney *U* test serves as a robust alternative for nonnormal distributions, ensuring appropriate statistical rigor.

#### Multivariate Analysis

Binary logistic regression will be performed to investigate the relationship between poor sleep hygiene (as the predictor) and cardiometabolic risk factors, while adjusting for potential confounders, such as age, gender, and socioeconomic status. Results will be presented as odds ratios with 95% CIs, with statistical significance defined as *P*<.05, allowing for a nuanced understanding of independent associations in the data.

### Potential Confounders

Data on various potential confounding factors will be collected, including sociodemographic factors such as education and occupation or work schedule; lifestyle habits such as coffee intake, diet, physical activity, and screen time; and psychological factors such as stress and anxiety or depression.

The 9-item Patient Health Questionnaire (PHQ-9) will be used to assess depression in participants [26], while the 7-item Generalized Anxiety Disorder (GAD-7) scale will be used to assess anxiety [27]. Physical activity will be assessed using the Global Physical Activity Questionnaire (GPAQ) [28]. For dietary assessment, the 24-hour recall method will be used. Caffeine (or tea) intake will be assessed by a self-structured questionnaire. Screen time of the participant will be measured by the Screen Time Questionnaire [29].

## Results

The study commenced in January 2025 with conceptualization. Institutional Ethics Committee approval was obtained in June 2025, followed by registration with the Clinical Trial Registry–India in August 2025. Data collection commenced in August 2025, with 53 participants enrolled as of May 5, 2026. Enhancements were made, following the peer-review suggestions and insights, to the study tools for better assessing confounding variables (GAD-7 for anxiety, PHQ-9 for depression, and GPAQ for physical activity). Data collection is ongoing, with efforts to ensure uniformity across the study population. Data collection is projected to be completed by January 2027, with final statistical analysis and submission of the results projected for March 2027 (Figure 2).

**Figure 2:**
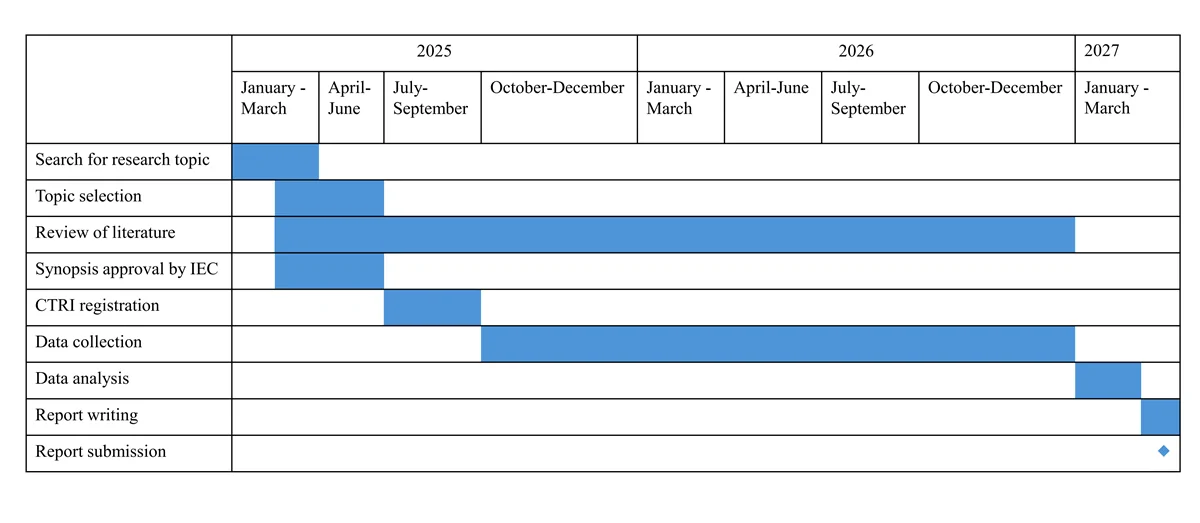
Execution schedule of past, ongoing, and projected study phases. CTRI: Clinical Trial Registry–India; IEC: Institutional Ethics Committee.

## Discussion

### Anticipated Findings

MetS is a prevalent health concern that has increased in recent years. Understanding the relationship between sleep hygiene and MetS may provide insights into how poor sleep hygiene contributes to cardiometabolic risk factors and may help inform strategies to prevent and mitigate cardiometabolic disease in the population. This study anticipates that poor sleep hygiene will be independently associated with higher rates of hypertension and hyperglycemia, consistent with findings from previous studies in urban populations. The study will contribute to the limited available literature on the association of poor sleep hygiene and MetS in the rural Indian population and may help identify appropriate measures for early intervention and prevention of the development of these risk factors.

Findings from this study will be disseminated to local health authorities and community stakeholders to guide targeted health interventions.

### Strengths

The study focuses on a door-to-door data collection approach, whereas most studies are hospital based, introducing selection bias. The approach of this study helps uncover undiagnosed cases, while also reducing selection bias. The methodology is further strengthened by a stratified random sampling design, helping ensure better generalizability of the findings of this study. The use of validated tools, such as the PSQI, IDRS, and SHI, will enhance the reliability, accuracy, and reproducibility of the data.

This study also incorporates real-time digital data entry to improve data integrity and reduce manual recording errors.

### Limitations

This cross-sectional, analytical study design inherently has certain limitations. First, recall bias will be a significant limitation, as data collection on sleep hygiene will be based on a self-answered questionnaire. Second, for the assessment of MetS, data on blood pressure, BMI, and FPG will be collected. Cholesterol and triglycerides will not be measured because of financial and logistical constraints. Although the presence of any 3 of 5 factors can be used for diagnosing an individual with MetS, and the study conducted by Brocato et al [1] was based on similar risk factors, a patient with only 2 of the 3 measured variables, while having one of the unmeasured variable (ie, cholesterol or triglyceride) abnormal, would still be labeled as an individual with no MetS, making this criteria for MetS diagnosis with the 3 variables highly stringent. With these limitations in mind, the study can still help obtain meaningful inferences from the data collected.

### Conclusions

The association between MetS and sleep hygiene warrants further investigation, particularly as poor sleep hygiene becomes an increasingly prominent health concern alongside the rising prevalence of MetS [30]. This study will contribute to developing a better understanding of the association of sleep hygiene and MetS, which will be fundamental in developing interventions and preventive strategies to help mitigate this issue.

## Data Availability

The datasets generated during this study will be available from the corresponding author on reasonable request.

## Abbreviations

DBP: diastolic blood pressure
FPG: fasting plasma glucose
GAD-7: 7-item Generalized Anxiety Disorder
GPAQ: Global Physical Activity Questionnaire
HDL: high-density lipoprotein
MetS: metabolic syndrome
PHQ-9: 9-item Patient Health Questionnaire
PSQI: Pittsburgh Sleep Quality Index
SBP: systolic blood pressure
SHI: Sleep Hygiene Index
